# Evaluation of the multimorbidity network and its relationship with clinical phenotypes in chronic obstructive pulmonary disease: The GALAXIA study

**DOI:** 10.1111/crj.13518

**Published:** 2022-06-22

**Authors:** Juan Marco Figueira‐Gonçalves, Rafael Golpe, Cristóbal Esteban, Miguel Ángel García‐Bello, Nagore Blanco‐Cid, Amaia Aramburu, Ignacio García‐Talavera, María Dolores Martín‐Martínez, Adrian Baeza‐Ruiz, Andrea Expósito‐Marrero

**Affiliations:** ^1^ Pneumology and Thoracic Surgery Service University Hospital Nuestra Señora de Candelaria Santa Cruz de Tenerife Spain; ^2^ University Institute of Tropical Diseases and Public Health of the Canary Islands University of La Laguna Santa Cruz de Tenerife Spain; ^3^ Pneumology Service University Hospital Lucus Augusti Lugo Spain; ^4^ Pneumology Service Galdakao‐Usansolo Hospital Galdakao Spain; ^5^ Health Services Research on Chronic Patients Network (REDISSEC) Galdakao‐Usansolo Hospital Barakaldo Vizcaya Spain; ^6^ Department of Clinical Psychology, Psychobiology and Methodology University of La Laguna (ULL), Guajara Campus San Cristobal de La Laguna Santa Cruz de Tenerife Spain; ^7^ Clinical Analysis Service University Hospital Nuestra Señora de Candelaria Santa Cruz de Tenerife Spain

**Keywords:** chronic bronchitis, comorbidities, COPD, exacerbation, network, phenotype

## Abstract

**Background:**

Chronic obstructive pulmonary disease (COPD) is a complex and heterogeneous condition, in which taking into consideration clinical phenotypes and multimorbidity is relevant to disease management. Network analysis, a procedure designed to study complex systems, allows to represent connections between the distinct features found in COPD.

**Methods:**

Network analysis was applied to a cohort of patients with COPD in order to explore the degree of connectivity between different diseases, taking into account the presence of two phenotypic traits commonly used to categorize patients in clinical practice: chronic bronchitis (CB^+^/CB^−^) and the history of previous severe exacerbations (Ex^+^/Ex^−^). The strength of association between diseases was quantified using the correlation coefficient Phi (ɸ).

**Results:**

A total of 1726 patients were included, and 91 possible links between 14 diseases were established. Although the four phenotypically defined groups presented a similar underlying comorbidity pattern, with special relevance for cardiovascular diseases and/or risk factors, classifying patients according to the presence or absence of CB implied differences between groups in network density (mean ɸ: 0.098 in the CB^−^ group and 0.050 in the CB^+^ group). In contrast, between‐group differences in network density were small and of questionable significance when classifying patients according to prior exacerbation history (mean ɸ: 0.082 among Ex^−^ subjects and 0.072 in the Ex^+^ group). The degree of connectivity of any given disease with the rest of the network also varied depending on the selected phenotypic trait. The classification of patients according to the CB^−^/CB^+^ groups revealed significant differences between groups in the degree of conectivity between comorbidities. On the other side, grouping the patients according to the Ex^−^/Ex^+^ trait did not disclose differences in connectivity between network nodes (diseases).

**Conclusions:**

The multimorbidity network of a patient with COPD differs according to the underlying clinical characteristics, suggesting that the connections linking comorbidities between them vary for different phenotypes and that the clinical heterogeneity of COPD could influence the expression of latent multimorbidity. Network analysis has the potential to delve into the interactions between COPD clinical traits and comorbidities and is a promising tool to investigate possible specific biological pathways that modulate multimorbidity patterns.

## Graphical abstract

The structure of the multimorbidity network of COPD patients can vary depending on underlying clinical phenotypes. This finding suggests that there are different biological mechanisms that influence the interactions between multimorbidities for different phenotypes.

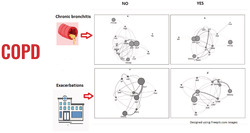

## 1 INTRODUCTION

Chronic obstructive pulmonary disease (COPD) is a highly complex and heterogeneous condition, in which other concomitant diseases contribute to symptom severity and cast a shadow on the patients' vital prognosis.^1^, ^2^, ^3^, ^4^, ^5^ Precision medicine has emerged in recent years as a new paradigm in the management of diseases. However, this approach is difficult to implement in patients with multimorbidity because of a lack of understanding on the complex relationships between different diseases.

Network analysis, a procedure designed to study complex systems, allows us to examine and draw links between distinct components.^6^, ^7^, ^8^, ^9^, ^10^ Such charts are made up of individual elements, called nodes, and a network of connecting lines, which represent their associations, thereby revealing the base system (a hierarchical, random, or scale‐free network). The network structure helps to identify highly connected nodes and offers great potential for its use in medicine.^7^

Furthermore, *cluster* analyses have been shown to identify subgroups of patients who share clinical and prognostic characteristics.^11^, ^12^, ^13^, ^14^, ^15^ This finding provides the basis for defining clinical phenotypes in COPD. Chronic bronchitis (CB) and frequent exacerbator (Ex) are phenotypes of recognized prognostic relevance in COPD.^16^, ^17^, ^18^ Furthermore, they are easily identified in clinical practice, in all healthcare settings. Consequently, some clinical practice guidelines have relied heavily on the classification of patients according to these phenotypes to propose different treatment regimens for COPD.^15^

Our hypothesis was that the biologic pathways that link the comorbidities would be different for the different clinical phenotypes of COPD and, as a consequence, the multimorbidity network of a patient with COPD would vary depending on such phenotypes. The objective of our study was to explore potential differences in the structure of the base system according to the thereby defined clinical phenotypes, applying the presence of CB and history of previous severe exacerbations as classification criteria. For this, the overall density of the network and the degree of connectivity of each disease (node) with the rest of the network were studied.

## 2 METHODS

### 2.1 Study population and settings

An observational, non‐interventional multicenter historic cohort study was performed involving three cohorts of patients with COPD from the University Hospital Nuestra Señora de Candelaria, Santa Cruz de Tenerife, the Galdakao‐Usansolo Hospital, Galdakao and the University Hospital Lucus Augusti, Lugo, all in Spain. These cohorts have been presented separately in previous, published works.^14^, ^18^, ^19^

Patients meeting the following criteria were included: follow‐up in a pneumology service, aged >40 years, active or former smokers with a pack‐year index (PYI) ≥ 10 and a forced expiratory volume in 1 s (FEV_1_)/forced vital capacity (FVC) ratio < 70% after administration of 400 μg of salbutamol. Exclusion criteria were the presence of chronic airflow obstruction without tobacco smoke exposure or with a PYI < 10 and chronic respiratory diseases other than COPD (e.g., interstitial lung disease and pneumoconiosis).

### 2.2 Variables

The variables used for analysis were age, sex, body mass index (BMI [kg m^−2^]) and history of tobacco consumption (PYI, current versus former smoker), assessed at first visit from each patient of each cohort.

Information about the following comorbid diseases was obtained: arterial hypertension (AHT), type 2 diabetes mellitus (T2DM), dyslipidaemia (DLP), sleep apnoea/hypopnoea syndrome (SAHS), obesity (defined as BMI ≥ 30 kg/m^2^), underweight (BMI < 18,5 kg/m^2^), atrial fibrillation (AF), ischaemic heart disease (IHD), heart failure (HF), cerebrovascular accidents (CVAs), peripheral arterial disease (PAD), chronic kidney disease (CKD), neoplasia (solid tumours, lymphoma, leukaemia), osteoporosis, heavy smokers (PYI > 50) and mood disorders (anxiety and depression). Each disease was confirmed by a comprehensive review of the computerized medical records, results of diagnostic procedures and disease‐specific therapies. Self‐reported diagnoses were not considered. The Charlson comorbidity index score^20^ was determined for each patient. Forced‐spirometry data following bronchodilation were recorded as FEV_1_%, FVC% and the FEV_1_/FVC ratio.

Moreover, the presence of CB (cough with sputum expectoration for at least 3 months a year during a period of at least 2 consecutive years) as well as the number of hospital admissions due to COPD exacerbation (episode of increasing respiratory symptoms, particularly dyspnoea, cough, sputum production and increased sputum purulence) in the 2‐year period prior to inclusion were recorded at first patient visit. The presence of CB was systematically registered in the databases of all the cohorts, and previous admissions were confirmed by review of hospital records. Based on the aforementioned, patients with or without symptoms of CB were assigned to the groups CB^+^ and CB^−^, respectively, and patients with or without previous hospitalizations for COPD exacerbation to the groups Ex^+^ and Ex^−^, respectively.

### 2.3 Data analysis

Multimorbidity network analysis was performed with the following objectives: (1) to evaluate network density by assigning patients to either of the two groups of clinical phenotypes (CB^+^/CB^−^ and Ex^+^/Ex^−^); (2) to analyse the degree of connectivity of a specific disease with the rest of the network for each of the mentioned phenotypic groups.

Depending on their distribution, quantitative variables were given as mean ± standard deviation (SD) or medians (interquartile ranges) and qualitative variables as frequencies (%). Quantile–quantile (QQ) plots were applied to graphically compare probability distributions. For quantitative variables, groups were compared by means of one‐way analysis if variance (ANOVA). The chi‐square test was applied for qualitative variables. In case of statistical significance between the three cohorts, paired differences between them were calculated and Holm's procedure applied to correct for multiple comparisons.

Point prevalence of disease was calculated by estimating proportions following the Clopper–Pearson method implemented in the binGroup package.^21^ Associations between diseases were analysed by chi‐square test or Fisher's test when the requirements for the former were not met. The degree of associations between diseases was quantified by relative risk (RR) estimation with 95% confidence intervals (CIs) as well as the correlation coefficient Phi (ɸ), proposed by Hidalgo et al.^9^ The RR of observing a pair of diseases in the same patient was calculated using the equation RRij = (Cij.N)/(Pi.Pj), where Cij stands for the number of patients with both diseases (ij), N is the total of patients and P indicates the number of patients with one of the two diseases. The correlation ɸ was calculated using the ɸ function of the psych package.^22^ The ɸ correlation coefficient is similar to the Pearson's correlation for dichotomous variables in its interpretation. The ɸ correlation coefficient ranges from −1 to +1, where +1 indicates perfect direct relationship, −1 indicates perfect inverse relationship and 0 indicates no relationship.

Out of the 16 evaluated diseases, 14 were finally used for analysis, as their prevalence was ≥3.5% in any of the subgroups (osteoporosis and underweight were excluded because its prevalence was <3.5%). To ensure homogeneity of the subgroup size, bootstrap re‐sampling was used. A sample size of 400 was used for re‐sampling Ex^+^ and Ex^−^ and 800 for CB^+^ and CB^−^ (applying for each pair a value close to the size of the smallest group). For each of the bootstrap re‐samples, the correlation ɸ between each pair of combinations and their associated probabilities of ɸ, the RRs as well as the associated probabilities and the prevalence of each disease were calculated. The final result was a mean ɸ from at least 10 000 re‐samples. The correlation between diseases was considered significant when the 2.5 percentile of ɸ was >0. Total network density was calculated as the mean of all ɸ, with values ranging from −1 to 1. Differences in network connectivity and density were based on numerical parameters.

Associations between the different diseases were plotted for each group. Node size was represented in proportion to the prevalence of the disease, and connecting lines between two nodes were drawn when the association between the two diseases was statistically significant with a ɸ > 0. Each connecting line was given a width in proportion to the square of the ɸ coefficient.

Multivariable logistic regression analyses were performed in order to evaluate the predictors of being classified within the Ex^+^ or the CB^+^ groups using comorbidities as predictive variables. Given the number of subjects belonging to the CB^+^ group (898) and the EX^+^ group (418), these analyses adhered to the 10 events per predictive variable rule.^23^

### 2.4 Ethics

The study was approved by the Ethics Committee of the University Hospital Nuestra Señora de Candelaria (reference number CHUNSC_2019_64).

## 3 RESULTS

A total of 1726 patients were included. The baseline characteristics of the study population are given in Table 1. Participants were mostly men with a mean age of 68 ± 9.2 years; 27.6% were active smokers with a predicted FEV_1_ of 52.9 ± 17.1% after bronchodilation. The CB^+^ group comprised 52% of the patients; 25.6% of the patients of the former group compared with 22.7% of the CB^−^ group had required hospitalization in the 2 years prior to inclusion (*p* = 0.176). Also, 55% of the patients in the Ex^+^ presented with mucus hypersecretion, compared to 51% of Ex^−^ patients (*p* = 0.172). Hospital admission for COPD exacerbation in the 2 years before inclusion had been necessary for 24.2% of the participants, regardless in which of the three hospitals. As to diseases, CB^−^ patients had a higher prevalence of AHT and HF than CB^+^ patients; the latter presented a higher smoking load (Table S1). AF, HF and CVA were more prevalent in Ex^+^ than in Ex^−^ patients (Table S2).

**TABLE 1:** Baseline characteristics of all included patients with COPD

	Overall	HGU	HUNSC	HULA	*p* value	
Patients (*n*)	1726	506	439	781		
Age (years)	68.4 ± 9.2	68.2 ± 8.1	69 ± 9.8	68 ± 9.4	0.20	
Sex, men	1534 (88.9)	490 (96.8)	346 (78.8)	698 (89.4)	<0.001	ABC
BMI	28.2 ± 5.3	28.2 ± 4.4	28.3 ± 6.0	28.2 ± 5.3	0.99	
Current smoker	476 (27.6)	122 (21.1)	154 (35.1)	210 (26.9)	<0.001	AC
PYI; n_HULA_ = 694	50 (35–63)	45 (30–60)	40 (30–60)	50 (40–80)	<0.001	ABC
PYI > 50; n_HULA_ = 694	652 (39.8)	199 (39.3)	128 (29.2)	325 (46.8)	<0.001	ABC
Charlson index	2.2 ± 1.5	2.4 ± 1.4	2.4 ± 1.5	2.0 ± 1.3	<0.001	BC
FEV_1_ (%)	52.9 ± 17.1	55.0 ± 13.1	55.3 ± 20.2	50.1 ± 17.1	<0.001	BC
FVC (%); n_HULA_ = 759	77.3 ± 17.9	76.43 ± 14.3	82.89 ± 21.2	74.69 ± 17.3	<0.001	AC
FEV_1_/FVC	51.0 ± 11.5	54.47 ± 9.4	51.08 ± 11.8	48.7 ± 12.1	<0.001	ABC
CB	898 (52)	368 (72.7)	147 (33.5)	383 (49)	<0.001	ABC
Previous hospitalization	418 (24.2)	132 (26.1)	90 (20.5)	196 (25.1)	0.10	
Comorbidities						
Obesity; n_HULA_ = 755	610 (35.9)	163(32.2)	169 (38.5)	278 (36.8)	0.103	
Underweight; n_HULA_ = 755	41 (2.4)	6 (1.2)	19 (4.3)	16 (2.1)	0.006	ABC
AHT; n_HULA_ = 279	624 (51.0)	191 (37.7)	290 (66.1)	143 (51.3)	<0.001	
T2DM	334 (19.4)	82 (16.2)	143 (32.6)	109 (14)	<0.001	AC
DLP; n_HGU_ = 0; n_HULA_ = 278	409 (57)	ND	295 (67.2)	114 (41)	<0.001	C
AF	235 (13.6)	66 (13)	89 (20.3)	80 (10.2)	<0.001	AC
ERC, n_HUNSC_ = 438	95 (5.5)	8 (1.6)	56 (12.8)	31 (4)	<0.001	ABC
SAHS	232 (13.4)	36 (7.1)	05 (23.9)	91 (11.7)	<0.001	ABC
HF	219 (12.7)	74 (14.6)	63 (14.4)	82 (10.5)	0.045	
IHD	201 (11.6)	31 (6.1)	71 (16.2)	99 (12.7)	<0.001	AB
CVA	108 (6.3)	38 (7.5)	32 (7.3)	38 (4.9)	0.094	
PAD	177 (10.3)	47 (9.3)	49 (11.2)	81 (10.4)	0.632	
MD	183 (10.6)	62 (12.3)	72 (16.4)	49 (6.3)	<0.001	AC
Np	109 (6.3)	0 (0)	48 (10.9)	61 (7.8)	<0.001	AB

*Note*: Data are presented as *n* and *n* (%), mean ± SD or median (interquartile range). A stands for significant differences between HGU and HUNSC, B for significant differences between HGU and HULA and C for significant differences between HUNSC and HULA.

Abbreviations: AF, atrial fibrillation; AHT, arterial hypertension; CB, presence of chronic bronchitis; CKD, chronic kidney disease; CVA, cerebrovascular accident; DLP, dyslipidaemia; FEV_1_ (%), percent‐predicted forced expiratory volume in 1 s; FVC (%), percent‐predicted forced vital capacity; HF, heart failure; HGU, Galdakao‐Usansolo Hospital; HULA, University Hospital Lucus Augusti; HUNSC, University Hospital Nuestra Señora de Candelaria; IHD, ischaemic heart disease; MD, mood disorder; ND, no data (not available); Np, neoplasia; PAD, peripheral arterial disease; PYI, pack‐year index; SAHS, sleep apnoea/hypopnoea syndrome; T2DM, type 2 diabetes mellitus.

**FIGURE 1:**
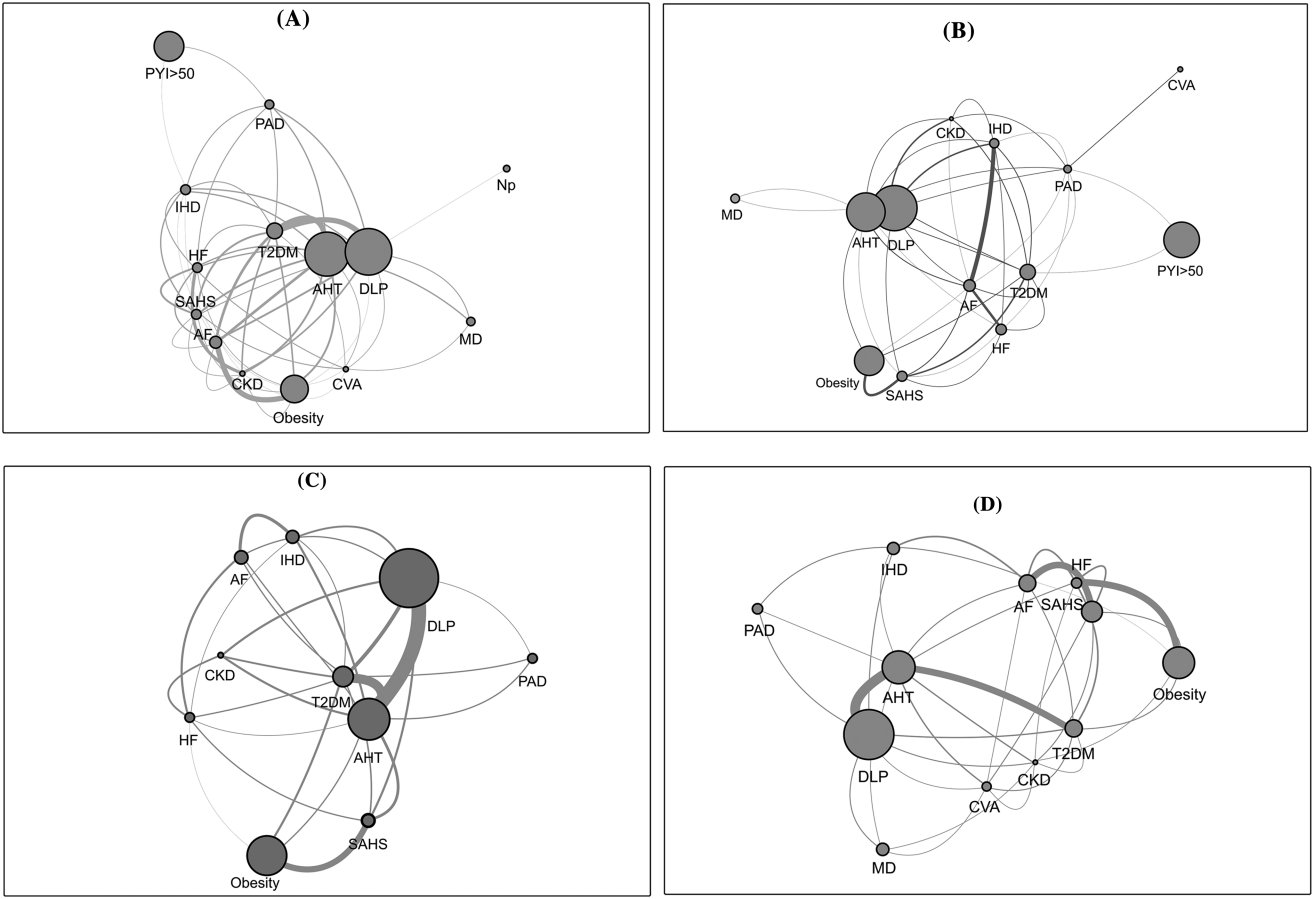
Multimorbidity network morphology and density according to clinical phenotypes in chronic obstructive pulmonary disease (COPD). (A) Patients without chronic bronchitis; (B) patients with chronic bronchitis; (C) patients without previous severe exacerbations; (D) patients with previous severe exacerbations. Node size is represented in proportion to the prevalence of the disease. Connecting lines between two nodes stand for a statistically significant association between the two diseases and for ɸ > 0. The width of each connecting line is proportional to the square of the ɸ coefficient. AF, atrial fibrillation; AHT, arterial hypertension; CKD, chronic kidney disease; CVA, cerebrovascular accident; DLP, dyslipidaemia; HF, heart failure; IHD, ischaemic heart disease; MD, mood disorder; Np, neoplasia; PAD, peripheral arterial disease; PYI, pack‐year index; SAHS, sleep apnoea/hypopnoea syndrome; T2DM, type 2 diabetes mellitus

Network analysis comprised the 91 possible links between 14 diseases. Network density values, assessed by mean ɸ, were modest (i.e., below 0.1) for all study groups, but differences were found between mutually exclusive phenotype‐defined groups. Analyses based on grouped data revealed the largest differences were found between the CB phenotypes. The highest density was found in the CB^−^ group (mean ɸ = 0.098) compared with the CB^+^ group (mean ɸ = 0.050). Differences in density network between the two Ex groups were smaller, with a mean ɸ = 0.082 in the Ex^−^ group and a mean ɸ = 0.072 in the Ex^+^ group (Figure 1).

**FIGURE 2:**
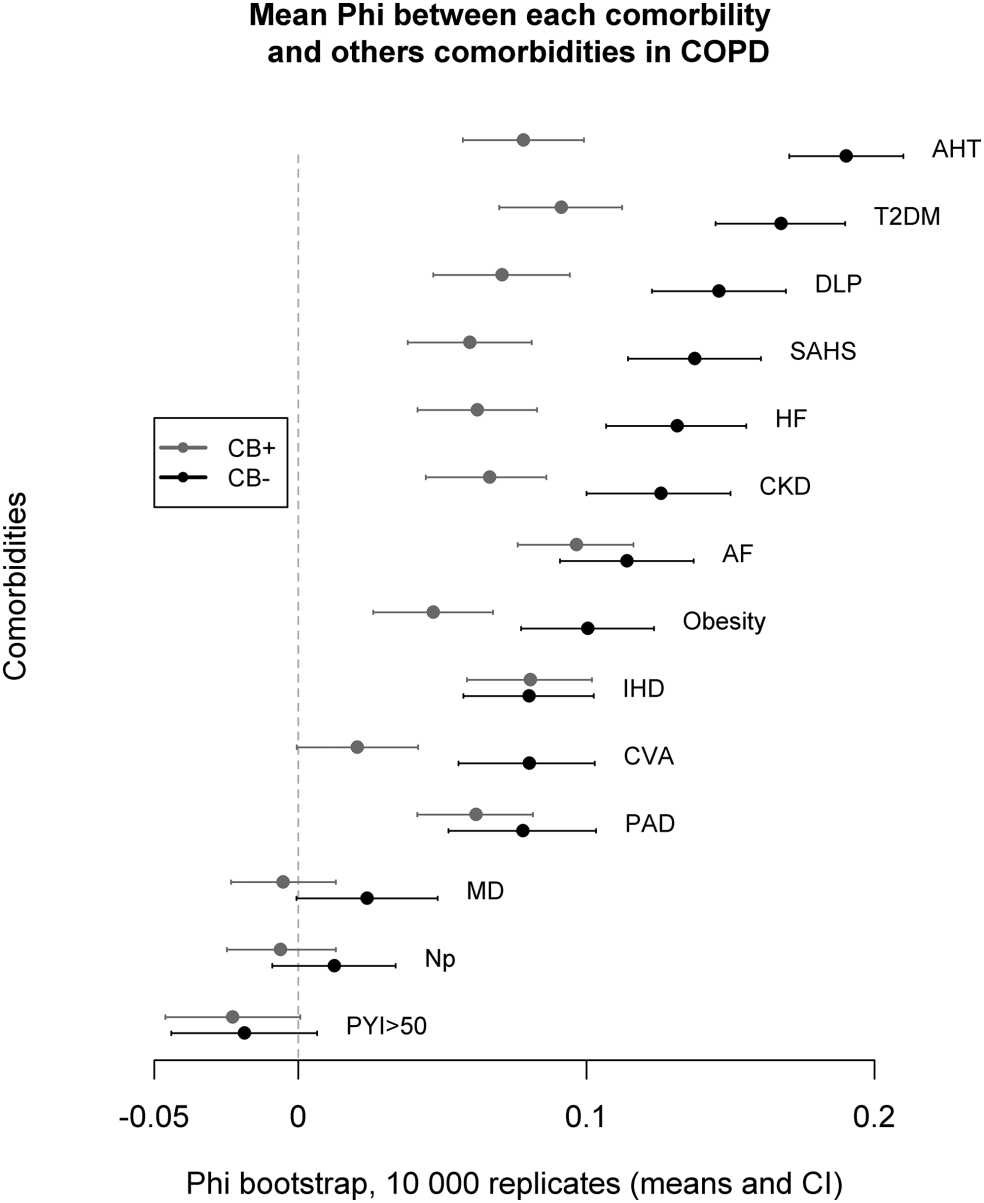
Degree of connectivity of each node (disease) with the rest of the network in accordance with the phenotype groups of chronic bronchitis (CB^+^ vs CB^−^). There are differences between both groups; the degree of connectivity is higher (higher value of Phi) for most of the nodes in the CB^−^ group. AF, atrial fibrillation; AHT, arterial hypertension; CKD, chronic kidney disease; CVA, cerebrovascular accident; DLP, dyslipidaemia; HF, heart failure; IHD, ischaemic heart disease; MD, mood disorder; Np, neoplasia; PAD, peripheral arterial disease; PYI, pack‐year index; SAHS, sleep apnoea/hypopnoea syndrome; T2DM, type 2 diabetes mellitus

**FIGURE 3:**
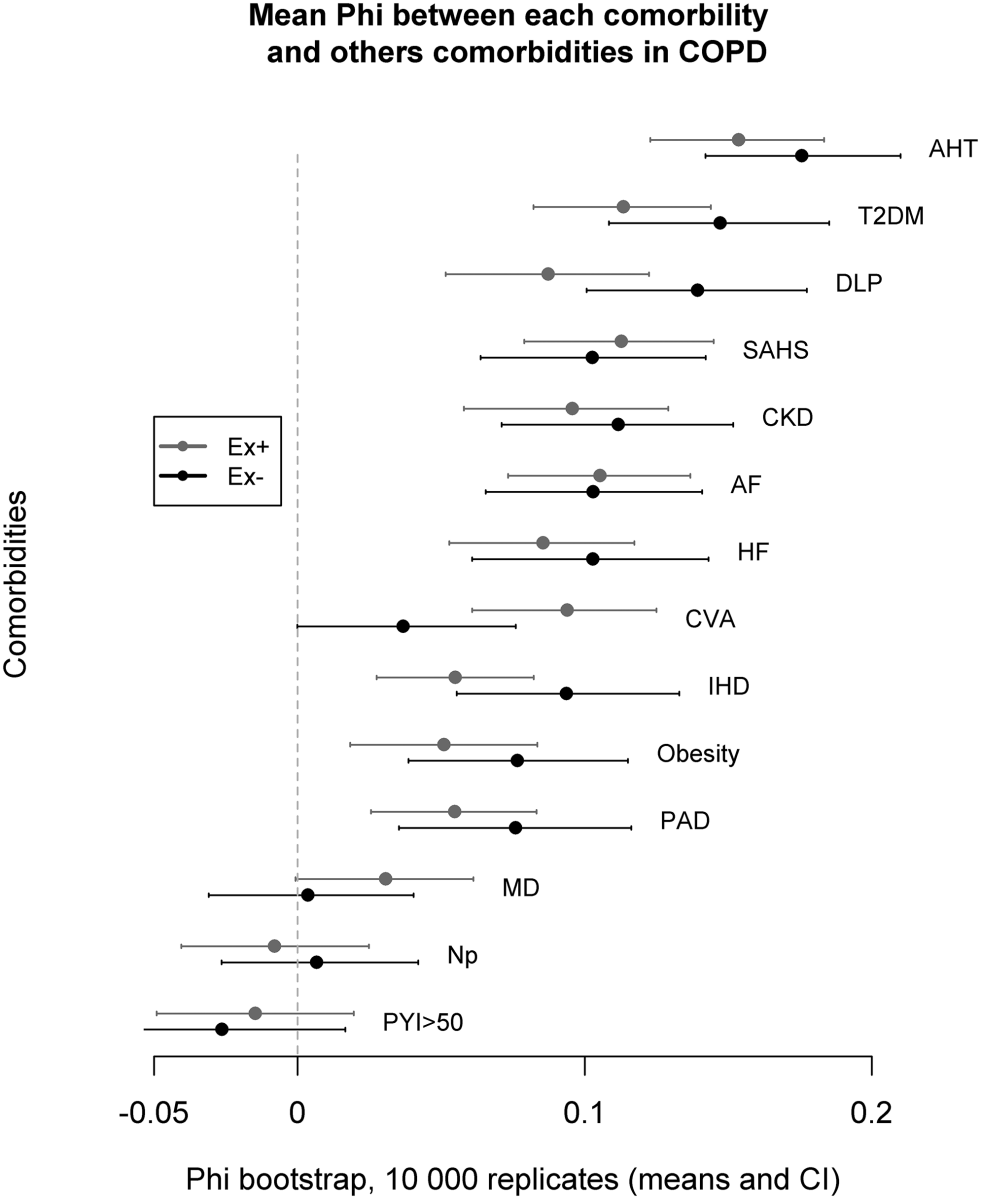
Degree of connectivity of each node (disease) with the rest of the network, according to the exacerbation background, that is, having suffered (Ex^+^) or not (Ex^−^) severe exacerbations in the past. Differences were not significant (Phi values between the two groups overlap). AF, atrial fibrillation; AHT, arterial hypertension; CKD, chronic kidney disease; CVA, cerebrovascular accident; DLP, dyslipidaemia; HF, heart failure; IHD, ischaemic heart disease; MD, mood disorder; Np, neoplasia; PAD, peripheral arterial disease; PYI, pack‐year index; SAHS, sleep apnoea/hypopnoea syndrome; T2DM, type 2 diabetes mellitus

The connectivity of a specific node (a disease) with the rest of the network varied according to the analysed phenotype (Figure 2). In the CB^−^ group, the nodes related to cardiovascular risk factors (AHT, DLP, T2DM), SAHS, HF and CKD displayed the largest connectivity within the network. In the CB^+^ group, the most intensely connected nodes were T2DM, AF and HF. In contrast, there was no difference in the degree of connectivity of disease between the groups Ex^+^ and Ex^−^ (Figure 3). On both Ex groups, the nodes AHT, T2DM, SAHS and AF displayed the largest connectivity within this network.

## 4 DISCUSSION

The conclusions drawn from this study are as follows: (1) Multimorbidity network densities vary with the underlying clinical features within COPD patients. (2) The degree of connectivity of a specific disease varies according to the phenotype group it belongs to.

Morbidity associated with COPD increase the risk of mortality. The evaluation of comorbid conditions is an important subject of clinical research in this disease. Mortality increases with the number of long‐term diseases in a given patient, but there is more than just the cumulative number of multimorbidities, and previous studies suggest that in the general population, some disease clusters are more strongly associated with all‐cause mortality relative to the number of coexisting diseases alone.^24^ A cluster that includes COPD, diabetes and cardiovascular diseases seems to have the greatest impact on mortality.^24^ Therefore, it is desirable to delve into the relationships between comorbid conditions and COPD.

Network analysis can provide new insights into the pathobiology of diseases. Hidalgo et al.^9^ investigated connections between multimorbidities in a Medicare database and found that diseases with high connectivity within the network were associated with decreased survival compared with ‘less connected’ comorbidities. The comorbidity network behaves analogous to airport networks; with their dissimilarly connected airports, air traffic will suffer mainly when delays affect centres with more traffic.^5^ It could be hypothesized that network disruption could be achieved through highly connected diseases.^5^

Our aim was to identify possible different patterns within the COPD comorbidity network that could increase our understanding of the complex relationships between multimorbid diseases. This could potentially serve as the basis for personalized follow‐up or therapeutic strategies, that is, intervening on the nodes that could lead to destabilization of the network. Given the heterogeneity of COPD, it is plausible that the biological pathways leading to adverse clinical outcomes may differ between patients with different clinical characteristics. We decided to analyse the influence of two phenotypic traits (chronic sputum production and frequent severe exacerbations) on the structure of the comorbidity network. These traits were selected because of their recognized prognostic relevance and also because they are easily recognizable in all the clinical settings where COPD patients are managed, including primary care medicine.

Although the resulting four groups showed a similar underlying pattern, with special relevance for AHT, DLP and T2DM, the morphology and density of the comorbidity network varied with the phenotype group. It should be noted that the density of comorbidity networks was not high in any of the study groups, but we found differences between the different phenotypes. The resulting network of the CB^−^ group had a higher density than that of the CB^+^ patients, and cardiovascular risk factors (AHT, T2DM, DLP), as well as SAHS, were highly connected in this group. If network alteration could be achieved by acting on highly connected conditions, the modification of these risk factors in the CB^−^ group of COPD patients could have a greater impact on the integrity of the system than in CB^+^ patients. Although the overall network of the latter showed a lower density, we observed that the major determinants of network integrity were the nodes representative of some established heart diseases (AF and IHD), rather than the cardiovascular risk factors. Anderson et al. described an increase in left ventricular mass in normoxemic COPD patients regardless of their history of AHT.^25^ This finding points to a potential underlying mechanism beyond classical cardiovascular risk factors that would favour the development of heart disease. A persistent, low‐grade inflammatory state might be this underlying mechamism.^26^, ^27^, ^28^ Because low grade systemic inflammation might be higher in subjects with CB,^29^ it is plausible that acting on nodes that represent classic cardiovascular risk factors such as AHT or T2DM provides a lower benefit in CB^+^ than in CB^−^ patients.

On the contrary, we did not find a significantly different behaviour of the comorbidity network when classifiying patients into groups regarding the incidence of exacerbations. Cardiovascular risk factors (AHT, T2DM and DLP) were also the most connected nodes, and therefore, acting on them should theoretically have a greater impact on the integrity of the network and, consequently, offer the greatest clinical benefit. However, our findings do not support the possibility that this phenotypic trait (frequent severe exacerbations) by itself impact the relationship between comorbidities. This is counterintuitive but could be explained by a survival bias effect due to the study design. Exacerbations that require hospitalization are major determinants of a poor clinical course and are associated with an increase in mortality that multiplies with each episode.^30^, ^31^, ^32^ As this was a study that retrospectively defined the frequent exacerbator phenotype, only patients who survived the exacerbations were included. Therefore, we could have selected patients with milder disease or with a lower comorbidity burden, which could have biassed our results.

To our knowledge, this is the first study to analyse the multimorbidity network in COPD according to clinical phenotypes. Among the strengths of the study are its relatively large sample size and multicentre design. The differences in the characteristics of the patient between the different cohorts, far from being a limitation, allowed us to cover the heterogeneity of the population with COPD. On the other hand, the retrospective design is a significant limitation that could have biassed the results of the analysis based on the exacerbator phenotype, as previously mentioned. Also, we were unable to include some elements that could have further clarified the interactions within the network, such as markers of systemic inflammation. Further prospective studies including other potential modifiers of the network structure are warranted.

In conclusion, we have found that the structure of the multimorbidity network of COPD patients can vary depending on some underlying clinical phenotypes. This finding suggests that there are different biological mechanisms that influence the interactions between multimorbidities for different phenotypes. Additional studies, using network analysis, should be performed to further clarify the association between biomarkers, clinical phenotypes and multimorbidities and to identify those elements that can be modified to obtain greater disruption of the multimorbidity network in different groups of patients. This could possibly identify novel treatable traits and help design personalized treatment strategies in COPD.

## CONFLICT OF INTEREST

The authors declare no conflict of interest, directly or indirectly related to the manuscript, to disclose.

## ETHICS STATEMENT

The study was approved by the Ethics Committee of the University Hospital Nuestra Señora de Candelaria (reference number CHUNSC_2019_64). The study was conducted in accordance with the Declaration of Helsinki.

## AUTHOR CONTRIBUTIONS

Juan Marco Figueira‐Gonçalves, Rafael Golpe and Cristobal Esteban performed the conception and manuscript design; data collection; data analysis and interpretation; and drafting, revising and approval of the manuscript. Miguel Ángel García Bello performed the conception and manuscript design; data analysis and interpretation; and drafting, revising and approval of the manuscript. Nagore Blanco‐Cid, Amaia Aramburu, Ignacio García‐Talavera, Maria Dolores Martín Martínez, Adrian Baeza‐Ruiz and Andrea Expósito‐Marrero did the data collection and drafting, revising and approval of the manuscript. All authors gave their approval to the final version of the manuscript and declare to have met the requirements for authorship. All authors have made substantial contributions to the intellectual content and manuscript design and drafting.

## Supporting information


**TABLE S1** Disease prevalence according to the presence of chronic bronchitis. Data are presented as n (%). AHT: arterial hypertension; T2DM: type 2 diabetes mellitus; DLP: dyslipidaemia; AF: atrial fibrillation; CKD: chronic kidney disease; SAHS: sleep apnoea/hypopnoea syndrome; HF: heart failure; IHD: ischaemic heart disease; CVA: cerebrovascular accident; PAD: peripheral arterial disease; MD: mood disorder; Np: neoplasia; PYI: pack‐year index.

**TABLE S2** Disease prevalence according to the criterion of previous hospitalisation for exacerbation. Data are presented as n (%). AHT: arterial hypertension; T2DM: type 2 diabetes mellitus; DLP: dyslipidaemia; AF: atrial fibrillation; CKD: chronic kidney disease; SAHS: sleep apnoea/hypopnoea syndrome; HF: heart failure; IHD: ischaemic heart disease; CVA: cerebrovascular accident; PAD: peripheral arterial disease; MD: mood disorder; Np: neoplasia; PYI: pack‐year index.

## Data Availability

Research data are not shared.
