# Supplementary material for: Evaluation of the multimorbidity network and its relationship with clinical phenotypes in chronic obstructive pulmonary disease: The GALAXIA study
Source: Clin Respir J. 2022 Jun 22;16(7):504–12. doi: 10.1111/crj.13518 (PMC9329016; doi:10.1111/crj.13518)
Supplement: Supplementary file 1 — TABLE S1 Disease prevalence according to the presence of chronic bronchitis. Data are presented as n (%). AHT: arterial hypertension; T2DM: type 2 diabetes mellitus; DLP: dyslipidaemia; AF: atrial fibrillation; CKD: chronic kidney disease; SAHS: sleep apnoea/hypopnoea syndrome; HF: heart failure; IHD: ischaemic heart disease; CVA: cerebrovascular accident; PAD: peripheral arterial disease; MD: mood disorder; Np: neoplasia; PYI: pack‐year index. [file CRJ-16-504-s002.docx]

TABLE S1 Comorbidity prevalence according to the presence of chronic mucus hypersecretion

| **Variables** | **CB^-^** | **CB^+^** | **p-value** |
| --- | --- | --- | --- |
| **Patients (n)** | 828 | 898 |  |
| Obesity | 297 (36.3) | 313 (35.5) | 0.763 |
| **AHT** | **291 (49.8)** | **187 (31)** | **<0.001** |
| T2DM | 166 (20) | 168 (18.7) | 0.52 |
| DLP | 188 (41.7) | 120 (45.1) | 0.414 |
| AF | 116 (14) | 119 (13.3) | 0.698 |
| **CKD** | **63 (7.6)** | **32 (3.6)** | **<0.001** |
| SAHS | 125 (15.1) | 107 (11.9) | 0.062 |
| HF | 103 (12.4) | 116 (12.9) | 0.821 |
| IHD | 101 (12.2) | 100 (11.1) | 0.54 |
| CVA | 55 (6.6) | 53 (5.9) | 0.593 |
| PAD | 87 (10.5) | 90 (10) | 0.801 |
| MD | 90 (10.9) | 93 (10.4) | 0.789 |
| Np | 62 (9) | 47 (8.9) | 1 |
| **PYI>50** | **291 (36.6)** | **361 (42.7)** | **0.014** |

Data are presented as n (%). AHT: arterial hypertension; T2DM: type 2 diabetes mellitus; DLP: dyslipidaemia; AF: atrial fibrillation; CKD: chronic kidney disease; SAHS: sleep apnoea/hypopnoea syndrome; HF: heart failure; IHD: ischaemic heart disease; CVA: cerebrovascular accident; PAD: peripheral arterial disease; MD: mood disorder; Np: neoplasia; PYI: pack-year index.
