# Supplementary material for: Evaluation of the multimorbidity network and its relationship with clinical phenotypes in chronic obstructive pulmonary disease: The GALAXIA study
Source: Clin Respir J. 2022 Jun 22;16(7):504–12. doi: 10.1111/crj.13518 (PMC9329016; doi:10.1111/crj.13518)
Supplement: Supplementary file 2 — TABLE S2 Disease prevalence according to the criterion of previous hospitalisation for exacerbation. Data are presented as n (%). AHT: arterial hypertension; T2DM: type 2 diabetes mellitus; DLP: dyslipidaemia; AF: atrial fibrillation; CKD: chronic kidney disease; SAHS: sleep apnoea/hypopnoea syndrome; HF: heart failure; IHD: ischaemic heart disease; CVA: cerebrovascular accident; PAD: peripheral arterial disease; MD: mood disorder; Np: neoplasia; PYI: pack‐year index. [file CRJ-16-504-s001.docx]

TABLE S2 Comorbidity prevalence according to the criterion of previous hospitalisation for exacerbation

| **Variables** | **Ex^–^** | **Ex^+^** | **p-value** |
| --- | --- | --- | --- |
| **Patients (n)** | 1308 | 418 |  |
| Obesity | 478 (36.9) | 132 (32.5) | 0.118 |
| **AHT** | **387 (42)** | **91 (34.3)** | **0.031** |
| T2DM | 252 (19.3) | 82 (19.6) | 0.931 |
| DLP | 242 (42) | 66 (46.8) | 0.349 |
| **AF** | **162 (12.4)** | **73 (17.5)** | **0.011** |
| CKD | 75 (5.7) | 20 (4.8) | 0.535 |
| SAHS | 185 (14.1) | 47 (11.2) | 0.153 |
| **HF** | **124 (9.5)** | **95 (22.7)** | **<0.001** |
| IHD | 151 (11.5) | 50 (12) | 0.885 |
| **CVA** | **70 (5.4)** | **38 (9.1)** | **0.008** |
| PAD | 130 (9.9) | 47 (11.2) | 0.501 |
| MD | 130 (9.9) | 53 (12.7) | 0.135 |
| Np | 75 (8) | 34 (11.9) | 0.06 |
| PYI>50 | 486 (38.9) | 166 (42.6) | 0.22 |

Data are presented as n (%). AHT: arterial hypertension; T2DM: type 2 diabetes mellitus; DLP: dyslipidaemia; AF: atrial fibrillation; CKD: chronic kidney disease; SAHS: sleep apnoea/hypopnoea syndrome; HF: heart failure; IHD: ischaemic heart disease; CVA: cerebrovascular accident; PAD: peripheral arterial disease; MD: mood disorder; Np: neoplasia; PYI: pack-year index.
